# The Role of the Residue at Position 2 in the Catalytic Activity of AA9 Lytic Polysaccharide Monooxygenases

**DOI:** 10.3390/ijms24098300

**Published:** 2023-05-05

**Authors:** Yucui Liu, Wei Ma, Xu Fang

**Affiliations:** State Key Laboratory of Microbial Technology, Shandong University, No. 72 Binhai Road, Qingdao 266237, China

**Keywords:** AA9 lytic polysaccharide monooxygenases, key amino acids, steric hindrance, catalytic activity, mechanism

## Abstract

AA9 lytic polysaccharide monooxygenases (LPMOs) are copper-dependent metalloenzymes that play a major role in cellulose degradation and plant infection. Understanding the AA9 LPMO mechanism would facilitate the improvement of plant pathogen control and the industrial application of LPMOs. Herein, via point mutation, we investigated the role of glycine 2 residue in cellulose degradation by *Thermoascus aurantiacus* AA9 LPMOs (*Ta*AA9). A computational simulation showed that increasing the steric properties of this residue by replacing glycine with threonine or tyrosine altered the H-bonding network of the copper center and copper coordination geometry, decreased the surface charge of the catalytic center, weakened the *Ta*AA9-substrate interaction, and enhanced *Ta*AA9-product binding. Compared with wild-type *Ta*AA9, G2T-*Ta*AA9 and G2Y-*Ta*AA9 variants showed attenuated copper affinity, reduced oxidative product diversity and decreased substrate Avicel binding, as determined using ITC, MALDI-TOF/TOF MS and cellulose binding analyses, respectively. Consistently, the enzymatic activity and synergy with cellulase of the G2T-*Ta*AA9 and G2Y-*Ta*AA9 variants were lower than those of *Ta*AA9. Hence, the investigated residue crucially affects the catalytic activity of AA9 LPMOs, and we propose that the electropositivity of copper may correlate with AA9 LPMO activity. Thus, the relationship among the amino acid at position 2, surface charge and catalytic activity may facilitate an understanding of the proteins in AA9 LPMOs.

## 1. Introduction

Until recently, the energy crisis and ecological environmental pollution have remained major challenges for sustainable global development [1,2]. Cellulose is one of the most abundant and widespread renewable resources, and it plays a critical role in the industrial production of biofuels and high-value chemical products [3]. However, the complex crystalline regions of cellulose still hinder effective and rapid conversion for cellulase degradation [1,4]. AA9 (Auxiliary Activity Family 9, formerly glycoside hydrolases 61(GH61)) lytic polysaccharide monooxygenases (LPMOs) can deconstruct the crystalline regions of cellulose through oxidative cleavage, which is a breakthrough in cellulose degradation and significantly enhances cellulose conversion efficiency via synergy with cellulase [5,6,7]. Therefore, AA9 LPMOs have excellent potential application value in cellulose exploitation and utilization as second-generation biofuels. Elucidation of AA9 LPMOs’ enzymatic mechanism is expected to facilitate enzymatic improvement and accelerate industrial application.

LPMOs are copper-dependent metalloenzymes, and copper plays a key role in the LPMO-mediated oxidation–reduction reaction and glycosidic bond cleavage [8,9,10,11,12]. The LPMO active site is constructed using a copper ion that is coordinated by two conserved histidine residues, an N-terminal histidine (H1) and a further histidine, forming a T–shaped coordination geometry [6,7,9,10]. In AA9 LPMOs, the active site also contains a conserved tyrosine located in the axial direction relative to the copper ion that participates in a H-bonding network near the copper active site, affecting the affinity of the copper or substrate for the LPMO [4]. Data show that the conserved tyrosine and tryptophan residues could couple to the copper center, which prevents the copper center from inactivation during uncouple turnover [4,6,12]. In addition, some other amino acids were shown to contribute to the enzymatic activity, substrate binding and regioselectivity of AA9 LPMOs. For example, enzymatic activity was decreased or lost when Q151 was replaced with a glutamic acid, asparagine, or leucine residue, and some tyrosine residues, such as Y203 in *Ls*LPMO9A from *Lentinus similis*, may interact with cellulose, and thus are related to interactions with substrates [13,14]. However, the working mechanism and structure–activity relationships of AA9 LPMOs, including which essential amino acids regulate enzymatic activity and catalytic processes and the relationship between the surface charge around the copper center and enzymatic activity, remain largely unknown [3].

*Ta*AA9 that is from *Thermoascus aurantiacus* is a typical AA9 LPMO, and it is a classic synergetic enzyme used in commercialized cellulose degradation [15]. H1, H86 and Y175 constitute the active site of *Ta*AA9 [16]. In this study, we focused on the amino acid at position 2 of AA9 LPMOs, and by means of a combination of computational simulation and experimental analysis, we investigated its role in regulating the enzymatic activity and catalytic process of *Ta*AA9. We hypothesized that there may be a positive correlation between electropositivity around the copper center of AA9 LPMOs and enzymatic catalysis.

## 2. Results

### 2.1. The Effect of the Residue at Position 2 on the Stabilization of the Copper Ion of TaAA9

**Figure 1:**
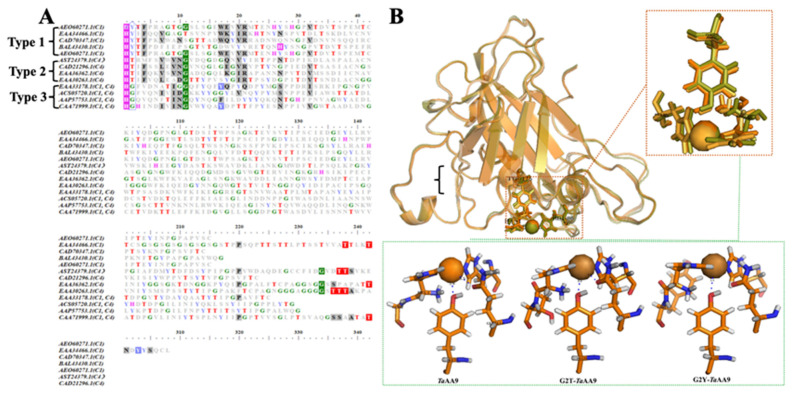
Stabilization of copper (center) in *Ta*AA9 and its mutants. (**A**) The identification of amino acid sequence alignments of 13 AA9 proteins with identified structures was performed with BioEdit 7.0. (**B**) The structural mode of the copper center in *Ta*AA9 and its mutants. The location of copper ions and the geometry of the copper center in *Ta*AA9 (PDB ID: 2yet) were visualized using PyMOL (top). Orange: *Ta*AA9; light orange: G2T-*Ta*AA9; yellow green: G2Y-*Ta*AA9. The H-bonding network around the copper ion in *Ta*AA9, G2T-*Ta*AA9 and G2Y-*Ta*AA9 visualized by PyMOL software 1.4.1 (green box), *Ta*AA9 (PDB ID: 2yet); the copper atoms are shown as orange spheres.

Through alignment of amino acid sequences of AA9 LPMO members, we found that the glycine, threonine and tyrosine residues are the most common residues at position 2 (Figure 1A), and these three residues have significantly different steric properties. In addition, the AA9 LPMOs are classified into three types: type 1, type 2, and type 3, and we noticed that glycine at position 2 was common in type 3 while threonine and tyrosine were more common in type 1 and type 2, respectively, as shown in Figure 1A. Meanwhile, the amino acid at position 2 was adjacent to H1, one of the two histidines that coordinate copper in the active site of LPMOs. It has been suggested that the steric properties of the amino acid in the active site of an enzyme may affect the architecture and physicochemical properties of the catalytic center and ultimately influence enzymatic activity [17,18]. Therefore, we first predicted the effect of the three amino acid residues at position 2 on AA9 LPMOs via bioinformation analysis, which suggests that the residue at position 2 may affect the physicochemical properties and substrate binding of AA9 LPMOs. As we know, *Ta*AA9 is one of the classic AA9 LPMOs, and it has a clear crystalline structure. To elucidate the possible effects of the amino acid at position 2 on the catalytic activity of AA9 LPMOs, we substituted wild-type glycine 2 (G2) of *Ta*AA9 with a threonine or tyrosine residue via site-directed mutagenesis. The resulting *Ta*AA9 mutants, namely, G2T-*Ta*AA9 and G2Y-*Ta*AA9, together with wild-type *Ta*AA9, were utilized in the following studies.

We first explored the effect of the residue at position 2 on the stabilization of the copper ion in *Ta*AA9. In the active site of AA9 LPMOs, the conserved tyrosine residue contributes to the formation of a H-bonding network near the copper center for copper stabilization [4]. As shown in Figure 1B, the Cu-O (Y175) distance of *Ta*AA9 increased from 2.9 Å to 3.0 Å and 3.5 Å when G2 was replaced with a threonine and tyrosine residue, respectively. In addition, we observed that compared with that in wild-type *Ta*AA9, the Cu-O (Q173) distance in G2T-*Ta*AA9 decreased from 3.5 to 2.8 Å, whereas in G2Y-*Ta*AA9, no H-bond with Q173 exists. These results suggested that the steric properties of the residue at position 2 affected the H-bonding network near the copper ion. It was reported that mutation of Q167 to A in *Mt*PMO3* resulted in the loss of the H bond between glutamine and tyrosine, leading to destabilization of copper [19]. Thus, we conjectured that the residue at position 2 may be related to the stability of copper, which is crucial for the enzymatic activity of *Ta*AA9. To evaluate this hypothesis, *Ta*AA9, G2T-*Ta*AA9 and G2Y-*Ta*AA9 recombinant proteins were prepared and identified using SDS–PAGE (as shown in Figure S1), and ITC was used to measure the binding affinity of Cu^2+^ to *Ta*AA9 and its mutants. The dissociation constants for Cu^2+^ to *Ta*AA9, G2T-*Ta*AA9 and G2Y-*Ta*AA9 were 1.45e^−6^ M, 1.59e^−6^ M and 12.6e^−6^ M, respectively, as shown in Figure S2. Compared with the previous data of the dissociation constants for Cu^2+^ to *Ta*AA9, we found that our result is different from the previous data in the reference (suggesting a KD tighter than 1 nM) [9]. We summarized the differences in the experimental method between the reference and this study (as shown in Table S1), but we could not explain why our results are different. However, we further measured the Km value of *Ta*AA9 that is similar to the data in the reference [20], as shown in Table S2. Therefore, these results can only be used to draw the relative differences between wild type and the mutants. This result indicates that replacing Gly-2 in *Ta*AA9 with a tyrosine residue decreased the affinity of Cu^2+^ for the copper center of *Ta*AA9. Therefore, we conclude that the residue at position 2 is an essential site affecting the H-bonding network around the copper center and plays a critical role in stabilizing the copper ion.

### 2.2. The Effect of the Residue at Position 2 on the Oxidative Products of TaAA9

**Figure 2:**
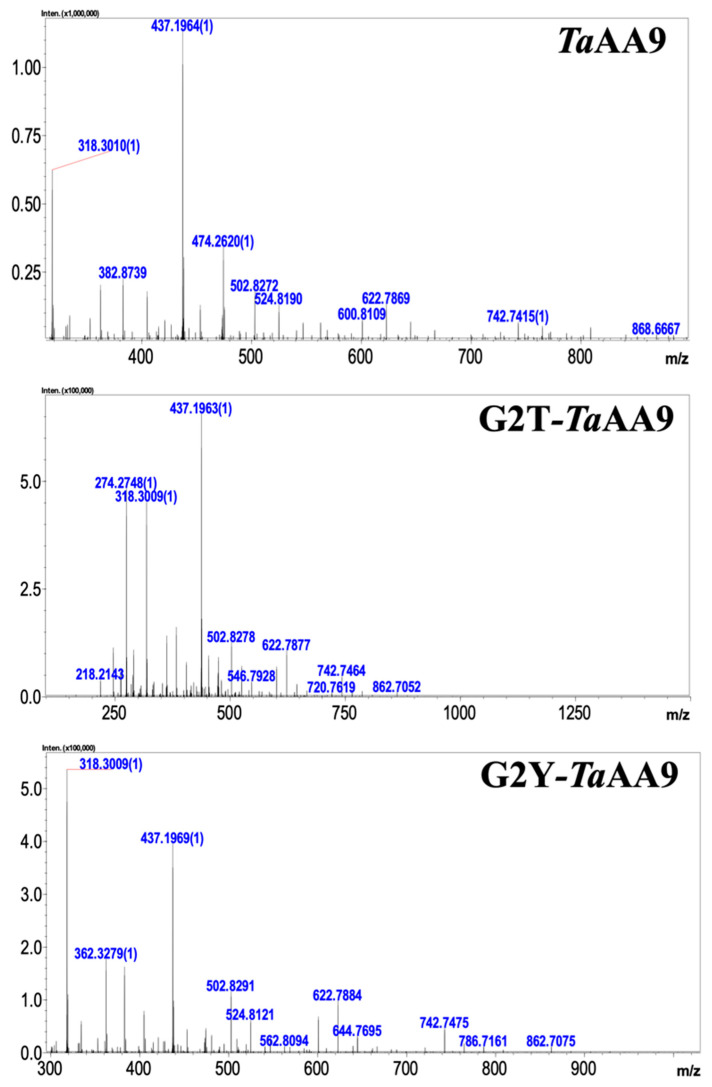
Analysis of oxidative products generated by *Ta*AA9 and its mutants. MALDI-TOF analysis of products generated by *Ta*AA9 and its mutants. The reactions containing 5 mg/mL PASC, 20 μM enzyme and 1 mM ascorbate were carried out in an incubator at 45 °C for 48 h and then analyzed by MALDI-TOF/TOF MS.

Vu et al. proposed that the geometry of a copper ion affects the oxidative cleavage sites of LPMOs [21,22]. The structural data in Figure 1B show that the substitution of glycine 2 with a threonine or tyrosine residue in *Ta*AA9 caused the alteration of the first coordination sphere geometry of the copper center. Therefore, we considered whether the residue at position 2 might play a key role in determining the sites of the oxidative reaction catalyzed by *Ta*AA9. To this end, we detected the oxidative products released from PASC upon catalysis by *Ta*AA9, G2T-*Ta*AA9 and G2Y-*Ta*AA9 using MALDI-TOF/TOF MS. As shown in Table 1 and Figure 2, the degree of polymerization (DP) of the cello-oligosaccharide products released after *Ta*AA9 catalysis ranged from DP_2_ to DP_5_, whereas the DP_5_ products were absent among the products obtained via G2T-*Ta*AA9 and G2Y-*Ta*AA9 catalysis. Moreover, the products resulting from oxidation at the C1 position were detected only after *Ta*AA9 catalysis but not after G2T-*Ta*AA9 or G2Y-*Ta*AA9 catalysis. These results indicate that the types of products released after *Ta*AA9 catalysis were more diverse than those released after G2T-*Ta*AA9 and G2Y-*Ta*AA9 catalysis, which was especially significant for DP_4_ and DP_5_ products (Table 1). Therefore, the DP and the diversity of product types were reduced when G2 in *Ta*AA9 was replaced with tyrosine or threonine, suggesting that the steric properties of the residue at position 2 in *Ta*AA9 may affect the first coordination sphere geometry of the copper center, thus influencing the oxidative site.

### 2.3. The Effect of the Residue at Position 2 on Substrate Binding in TaAA9

**Figure 3:**
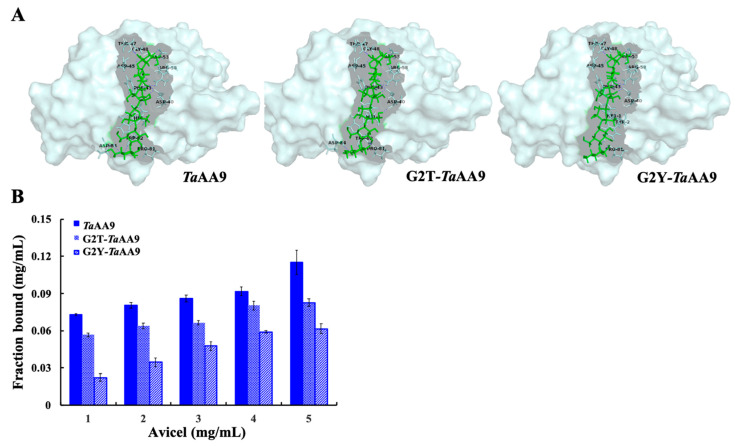
Analysis of the binding of *Ta*AA9 or its mutants to its substrates. (**A**) The binding mode of cellohexaose in *Ta*AA9 and its mutants as determined by MD simulation; green: cellohexaose, gray: *Ta*AA9 (PDB ID: 2yet) and mutants. This is presented in detail Figure S3, which shows (**B**) the extent of the binding of *Ta*AA9 and its mutants to Avicel. In the experiment, 0.2 mg of LPMO was incubated overnight with a series of concentrations of Avicel at 25 °C. The assay buffer was 10 mM HAc-NH4Ac, pH 5.0. The fraction of bound protein was determined as the difference between the initial concentration and the remaining concentration. In the control sample containing no Avicel, there was no difference in the control sample between the initial concentration and the remaining concentration.

Cellulose degradation by LPMOs is a dynamic process that involves substrate binding, enzymatic catalysis and product dissociation. The conformation of the enzyme–substrate complex is also a critical factor that affects the interaction between the enzyme and substrate, thus playing a key role in the catalytic process [23]. Therefore, we next investigated the effects of glycine, tyrosine and threonine residues at position 2 on substrate binding in the AA9 LPMOs. As shown in Figure 3A and Figure S3, compared with the wild-type *Ta*AA9, the glycosidic bonds of cellohexaose had a torsion from −1 to +2 when G2 in *Ta*AA9 was replaced by threonine or tyrosine, which led to the changes of the conformation of the G2T-*Ta*AA9-cellohexaose complex and G2Y-*Ta*AA9-cellohexaose complex. Additionally, as shown in Table S3, the H-bond distances in the G2T-*Ta*AA9-cellohexaose complex and G2Y-*Ta*AA9-cellohexaose complex were changed compared with wild-type *Ta*AA9, and significant differences were observed among D40, T47, W82 and D84. In addition, compared with that in wild-type *Ta*AA9, the H-bonding network in G2Y-*Ta*AA9 did not include W82 and D84, whereas Y2 formed a new H bond with the substrate. Moreover, binding free energy decreased when G2 in *Ta*AA9 was replaced by threonine or tyrosine, as shown in Table S4. These results suggested that W82 and D84 in *Ta*AA9 may be key sites that contribute to substrate binding. Previous research has shown that W82 in *Tf*AA10 (*Thermobifida fusca* AA10) is important for substrate binding [24], consistent with our current results. Moreover, our results implied that an increase in the steric hindrance of residues at position 2 may decrease the binding force and consequently reduce the binding ability of *Ta*AA9 to the substrate. To confirm the above conclusion, Avicel was used as a model to characterize the binding of wild-type and mutant *Ta*AA9 to the substrate. As shown in Figure 3B, the amount of bound protein was reduced when G2 in *Ta*AA9 was replaced with a tyrosine or threonine residue. The adsorption constants of *Ta*AA9, G2T-*Ta*AA9 and G2Y-*Ta*AA9 were 0.13 ± 0.008, 0.1 ± 0.007 and 0.087 ± 0.005, respectively, which were calculated by fitting the data to a Langmuir adsorption isotherm using Origin 8 software. The binding abilities of *Ta*AA9, G2T-*Ta*AA9 and G2Y-*Ta*AA9 to the substrate decreased in that order, which is consistent with our hypothesis above. Therefore, these data suggest that the glycine2 residue in *Ta*AA9 mediates H-bonding interactions with the substrate, which subsequently affects the conformation of the enzyme–substrate complex, ultimately influencing substrate binding.

Furthermore, the previous studies suggested that the aromatic residues could orient the oxidative site by slightly tuning the effect of oxidation of the copper ion toward the C1 or C4 glycosidic position [23,25]. Therefore, the effect of the residue at position 2 on the conformation of the enzyme–substrate complex by affecting W82 in *Ta*AA9 may be another reason for the different types of oxidative products generated when glycine2 was mutated. By combining the data shown in Figure 2 and Figure 3A and Table 1, we suggest that the residue at position 2 in *Ta*AA9 could interfere with the oxidative cleavage site by affecting the first coordination sphere geometry of the copper center and the conformation of the enzyme–substrate complex.

### 2.4. The Effect of the Residue at Position 2 on Enzymatic Catalysis of TaAA9

**Figure 4:**
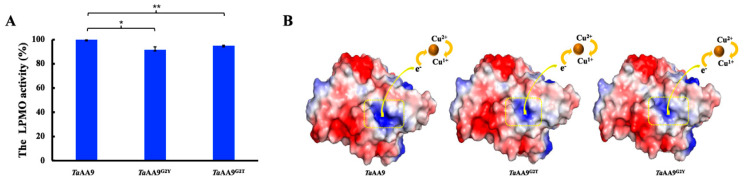
The enzymatic catalysis of *Ta*AA9 and its mutants. (**A**) The LPMO activity of *Ta*AA9 and its mutants. The reaction mixture included equal volumes of 1.2 μM enzyme, 800 μM rPHP, 400 μM DHA, and citrate–phosphate buffer (pH 7.25). When the reaction ended, 50 μL of stop buffer (Na_2_CO_3_, pH 10.3) was added to each well, and the absorption at 545 nm was measured. The data are presented as the mean ± standard deviation: * *p* < 0.1 and ** *p* < 0.01. (**B**) The surface charge around the copper center of *Ta*AA9 (PDB ID: 2yet) and its mutants are visualized using PyMOL software 1.4.1. Blue: electropositivity; brown: copper ion; yellow square: copper center.

Next, we explored the effect of the residue at position 2 on the enzymatic catalysis of *Ta*AA9. It has been reported that the catalytic process of LPMO requires external electrons to mediate the redox reaction of the copper center, which is a key step in enzymatic catalysis [26,27]. The increase in electropositivity is beneficial to electrophilic reactions and accelerates electron transfer [28]. It is well-known that the copper center is the terminal of the electron transfer chain, which accepts electrons to activate an oxidation–reduction reaction. Therefore, we investigated the effect of the residue at position 2 on the surface charge of the copper center. As shown in Figure 4B, electropositivity was observed around the copper center of *Ta*AA9, whereas the electropositivity was reduced when mutating Gly-2 to tyrosine or threonine. We hypothesized that the substitution of glycine 2 with a threonine or tyrosine residue in *Ta*AA9 resulted in a decrease in the degree of electropositivity around the copper center and may affect the oxidation–reduction potential between the cooper center and electron donor, which may consequently slow down enzymatic catalysis. As shown in Figure 4A, compared with that of wild-type *Ta*AA9, the LPMO activities of G2T-*Ta*AA9 and G2Y-*Ta*AA9 were decreased by 5% and 8.5%, respectively, along with the decrease in the degree of electropositivity around the copper center. We also observed a similar trend in other AA9 members from *Trichoderma reesei* QM6A and another AA9 LPMO, as shown in Figure S4. Considering the results as a whole, we propose that the electropositivity around the copper center of AA9 LPMOs may have a positive correlation with enzymatic catalysis, providing a possible explanation as to why the surface charge of the copper center of LPMOs is associated with enzymatic catalysis [3].

### 2.5. The Effect of the Residue at Position 2 on Product Dissociation from TaAA9

**Figure 5:**
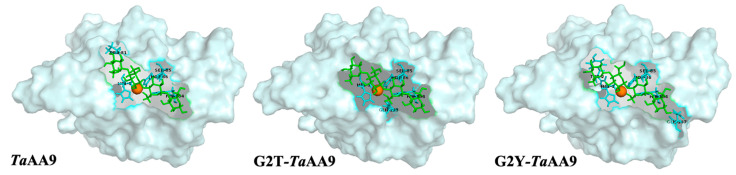
The binding mode of cellotetraose in *Ta*AA9A and its mutants visualized by PyMOL software 1.4.1. The mode of cellotetraose binding to *Ta*AA9 and its mutants by MD simulation, green: cellotetraose; gray: *Ta*AA9 (PDB ID: 2yet) and mutants; blue: amino acids.

Finally, we explored the effect of the residue at position 2 on the dissociation of the product from *Ta*AA9. As shown in Figure 2, cellotetraose (m/z 688.74, nonoxidative oligosaccharide) is one of the products released from PASC. Meanwhile, a capillary electrophoresis (CE) analysis found that the cellotetraose could bind with *Ta*AA9, as shown in Figure S5. Therefore, cellotetraose was used as a ligand to measure the interaction between *Ta*AA9 and its product by molecular simulation, as shown in Figure 5. The results indicate that the conformation of the *Ta*AA9–cellotetraose complex may be affected when G2 in *Ta*AA9 is replaced by threonine or tyrosine. The MD simulation results indicate that the binding free energy between *Ta*AA9 and cellotetraose was increased when G2 was replaced with a Thr or Tyr residue, as shown in Table S4. The enhanced interaction between the enzyme and product is not beneficial for release of the product from the enzyme, which may decrease the performance in the next catalytic cycle, and thus slow cellulose degradation. Therefore, we speculated that the residue at position 2 may be involved in the interaction between *Ta*AA9 and its product, thus affecting the dissociation of the product, which may be one of the ways the residue at position 2 regulates the catalytic activity of *Ta*AA9 in cellulose degradation. Consistently, we also found that the cellulose degradation of LPMO decreased when enzyme–product interactions increased in other AA9 LPMOs samples, as shown in Figure S6. Upon considering the data in Figure 3 and Figure 5 together, we believe that the residue at position 2 is the key site that mediates the interaction between the enzyme and substrate and exhibits different interaction strengths on the substrate and product, and which plays a key role in regulating the catalytic activity of LPMO.

### 2.6. The Effect of the Residue at Position 2 on the Synergetic Activity of TaAA9

**Figure 6:**
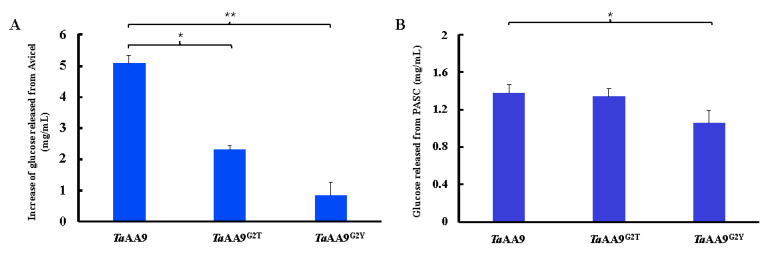
Glucose released by cellulase and *Ta*AA9 or its mutants. (**A**) Glucose released from Avicel by cellulase and *Ta*AA9. (**B**) Glucose released from PASC by cellulase and *Ta*AA9. The reaction contained the cellulase and β-glucosidase (1:1 ratio) in 50 mM sodium citrate at pH 4.8. The glucose content was determined by HPLC. The data are presented as the mean ± standard deviation. * *p* < 0.1 and ** *p* < 0.01.

AA9 LPMOs play a synergetic role by disrupting the crystalline regions of cellulose, which exposes the chain ends of cellulose to cellulase and enhances cellulose degradation [29]. Therefore, we further compared the synergetic activities of *Ta*AA9, G2T-*Ta*AA9 and G2Y-*Ta*AA9 on cellulase in vitro. As shown in Figure 6A, the addition of *Ta*AA9, G2T-*Ta*AA9 and G2Y-*Ta*AA9 increased the amount of glucose released from Avicel and catalyzed by cellulase to different extents. The amount of glucose released upon *Ta*AA9 addition was 29.5% higher than that achieved only by cellulase, whereas G2T-*Ta*AA9 and G2Y-*Ta*AA9 addition increased glucose release by 13.4% and 4.8% relative to that released by cellulase only, respectively. Therefore, compared with that released upon *Ta*AA9 addition, the amount of glucose released from Avicel upon G2T-*Ta*AA9 and G2Y-*Ta*AA9 addition decreased by 12.4% and 19.0%, respectively. Similarly, the amount of glucose released from PASC when G2T-*Ta*AA9 and G2Y-*Ta*AA9 variants were added decreased by 2.4% and 23.5%, respectively, compared with that released when *Ta*AA9 was added, as shown in Figure 6B. Based on these results, we concluded that the synergetic activity of *Ta*AA9 decreased when G2 was replaced with a Thr or Tyr residue. In addition, we obtained similar results when other AA9 samples from *Trichoderma reesei* QM6A were added to the catalytic reaction, as shown in Figure S4. All of these data collectively indicate that the steric properties of the residue at position 2 play a key role in regulating the catalytic activity of AA9.

## 3. Discussion

Digging into the details of active site properties is an effective way to understand the mechanism of AA9 LPMO in cellulose degradation. In this study, we investigated the effect of amino acid residue at position 2 in *Ta*AA9 on enzymatic activity and catalysis. Our data show that the residue at position 2 of *Ta*AA9 may affect cellulose degradation by *Ta*AA9 through the following pathways: (1) The steric properties of the residue at position 2 contribute to the geometry of the copper center, resulting in the influence on the affinity and orientation of the copper, which affect the enzymatic activity and oxidative site. (2) The steric properties of the residue at position 2 contribute to the strength of the electropositivity around the copper center, which may affect the oxidation–reduction potential of the copper center, and thus regulate the enzymatic catalysis of *Ta*AA9. (3) The steric properties of the residue at position 2 affect substrate binding and product dissociation by mediating H-bond formation, which also affects the catalytic activity of *Ta*AA9. Furthermore, our study proposed that there may be a positive correlation between the enzymatic activity and the electropositivity around the electron transfer chain of AA9 LPMOs.

The geometry coordinate of the copper center of LPMOs is related to the copper oxidation state and stabilization [19]. It has been reported that the H1, H86, Q173 and Y175, which are conserved in the center of AA9 LPMOs, constitute the active site of *Ta*AA9, and these amino acids form the geometry coordinate of the copper [4,16]. H1 and H86 directly contribute to the formation of T-shape geometry for coordinating copper [16]. The tyrosine residue is axially coordinated to the copper, and the Cu-O (tyrosine) distances range from 2.5 to 3.0 Å [3]. Q173 is the key amino acid of the secondary coordination sphere of the copper ion, which affects the reactivity of exogenous ligands bound to the copper [16]. Our study on the structure of the *Ta*AA9 active site showed that the Cu-O (tyrosine) distance increased from 2.9 Å to 3.0 Å and 3.5 Å, and the H-bond interaction formed by Q173 was absent when G2 was replaced with tyrosine residues, as shown in Figure 1. Our data indicate that the increase in the steric hindrance of the residue at position 2 interferes with the H-bonding network near the copper, leading to the decreased affinity of copper. Therefore, the residue at position 2 is another key site contributing to the formation of the copper center structure and plays a critical role in the stabilization of the copper.

Research on the substrate binding of LPMOs involves a challenge to understand the catalytic mechanism in detail. Previous research has proposed that some loop regions that connect the β-strands of the sandwich core and aromatic residues may contribute to substrate binding and regioselectivity. Some studies also suggest that the H-bonding network affects the interaction between the enzyme and ligand, also playing a key role in oxygen activation [4,30]. Our data indicate that the steric hindrance of the residue at position 2 affects the conformation of enzyme–substrate complex of *Ta*AA9, thus influencing the ability and strength of *Ta*AA9 to bind to its substrate (as shown in Figure 3 and Table S4). However, the interaction between *Ta*AA9 and its catalytic product, cellotetraose, was enhanced when G2 was replaced with Thr and Tyr residues (shown in Table S4). These data suggest that the residue at position 2 is involved not only in the interaction between *Ta*AA9 and its substrates, but also in the binding of *Ta*AA9 and its products, suggesting an essential role of the residue at position 2 in regulating the catalytic efficiency of *Ta*AA9. The study provided a key residue site essential for the substrate binding of LPMOs.

According to the preference of the oxidative cleavage site, AA9 LPMOs are classified into three types: type 1, oxidative cleavage at C1 of the glycosidic unit; type 2, oxidative cleavage at C4 of the glycosidic unit; and type 3, oxidative cleavage at both C1 and C4 [22,23]. However, the underlying mechanisms behind the oxidative regioselectivity of LPMOs is still unclear. There are some hypotheses regarding the selection of oxidative site: the geometry coordinate of the copper center may be related to the determination of the oxidative cleavage sites of LPMOs; the aromatic residues orient the oxidative site by slightly tuning the effect of oxidation of the copper ion toward the C1 or C4 glycosidic position; the surface-exposed axial copper coordination site seems to exhibit different restrictions among the C1-oxidizing, C4-oxidizing and mixed C1/C4-oxidizing, which relate to the regioselectivity [31]. Our data show that the residue at position 2 of *Ta*AA9 is involved in regulating the geometry coordinate of the copper center, conforming the enzyme–substrate complex and mediating the interaction between the axial copper coordination site (Tyr175) and copper. Therefore, the residue at position 2 can affect the oxidative site bias of *Ta*AA9, as evidenced by the production of different oxidative products when G2 was replaced with threonine and tyrosine residues (as shown in Table 1). However, further investigation is required to elucidate its underlying molecular mechanisms.

LPMOs need electron donors to offer electrons and activate the redox reaction [32]. LPMOs can accept electrons from different sources, including small-molecule reductants (e.g., ascorbate), enzymes (e.g., CDH) and light-harvesting pigments (e.g., chlorophyll) [33,34]. Some previous studies suggest that the electron transfer pathway of LPMOs involves the direct coupling of the external electron with Cu^2+^ to carry out a reduction reaction, while some data suggest that electron transfer to Cu^2+^ occurs via long-range electron transfer from an electron donor to LPMOs by a series of amino acids [25,35,36]. In either case, the electron is transferred to the copper ion of the catalytic center. Therefore, the physicochemical properties of the copper center may be the critical factor to influence the enzymatic catalysis of LPMOs. We found that the copper center of LPMOs showed electropositivity, which may be beneficial for the enhancement of the oxidation–reduction potentials of the copper center to attract electrons. The relationship between enzymatic activities and the electropositivity of the copper center in different LPMOs showed a similar trend in which the enzymatic activities were reduced as the electropositivity of the copper center became weakened. We hypothesized that there may be a positive correlation between the enzymatic activities and the electropositivity of the copper center of different LPMOs. In summary, we demonstrated that the residue at position 2 is one of the critical sites for *Ta*AA9 enzymatic catalysis, and it affects *Ta*AA9 activities via multiple mechanisms.

## 4. Materials and Methods

### 4.1. Materials

Avicel PH-101 was used to prepare phosphoric acid swollen cellulose (PASC) according to a previously described method [37]. Avicel was obtained from Tokyo Chemical Industry (Japan).

### 4.2. Strains and Enzymes

Cellulase was obtained from *Trichoderma reesei* (*T. reesei*) T1 that was previously stored in the laboratory [38]. The *Ta*AA9 gene was synthesized by GENEWIZ Company (Suzhou, Jiangsu, China), and the G2T-*Ta*AA9 and G2Y-*Ta*AA9 mutations were constructed using a KOD-PLUS-Mutagenesis kit. *E*. *coli* BL21 (DE3) (TransGen Biotech, Beijing, China) (Table S5) cells were used as a recombinant expression host. The recombinant protein expression was induced by adding 0.6 mM isopropyl β-D-1- thiogalactopyranoside (IPTG) to the recombinant strains, which were cultured at 16 °C and 120 rpm for 16 h. *Ta*AA9, G2T-*Ta*AA9 and G2Y-*Ta*AA9 were purified using a GE metal affinity resin by AKTA pure (elution buffer: 250 mM imidazole-PBS, pH 7.25) and identified using SDS–PAGE. β-Glucosidase was obtained using previously published protocols [39] and purified by means of a Q-Sepharose FF column (bed volume, 20 mL). The His-SUMO tag was added before the His 1 position, which can be completely cut by SUMO protease without the residue of any SUMO amino acids. The method used to reconstitute the enzyme with copper was to mix the *Ta*AA9 or its mutant recombinant proteins with equal equivalents of Cu^2+^ at 4 °C for 1 h, and then the buffer was exchanged using a molecular sieve.

### 4.3. Sequence Alignment Assay and Structural Bioinformation Analysis

The amino acid alignment of 13 full-length AA9 proteins whose structures were published in the Protein Data Bank (PDB) database was performed using Bioedit7.0 software. The crystal structure of *Ta*AA9 was downloaded from the Protein Data Bank (PDB ID: 2yet), whereas the structural models of G2T-*Ta*AA9 and G2Y-*Ta*AA9 variants were predicted using Phyre 2 Protein Homology/Analogy Recognition Engine V2.0. PyMOL software 1.4.1 was used to visualize the protein structure and compute the surface charge and H bonds of the protein.

### 4.4. Copper Ion Affinity Assay

Isothermal titration calorimetry (ITC) was used to determine the copper ion affinity to *Ta*AA9, G2T-*Ta*AA9 and G2Y-*Ta*AA9, as previously described, with some modifications [40]. Briefly, 30 μM or 50μM LPMO was added to the reaction cell, with 0.6 mM or 0.99 mM Cu(NO_3_)_2_ in the syringe. The assay buffer was 20 mM MES buffer, pH 5.5. Aliquots of 4 μL were injected at 150 s intervals with a stirring speed of 750 rpm at 25 °C. The titrations were finished after 19 injections. The ITC data were analyzed using Microcal PEAQ-ITC analysis software 1.1.0.1262. The experiments were repeated twice independently.

### 4.5. Oxidative Reaction Product Assay

The reaction products were measured as previously described [41]. Briefly, the reaction mixture consisted of 10 mM HAc-NH4Ac (pH 5.0) containing 5 mg/mL PASC, 20 μM enzyme, and 1 mM ascorbate, which were incubated at 45 °C for 48 h. After centrifugation, the supernatant was collected and analyzed by MALDI-TOF/TOF MS (LCMS-IT-TOF, Shimadzu, Japan). The method of sample analysis was measured as previously described [8]. Then, 2 μL of the sample was injected into 50% acetonitrile. The flow rate of the mobile phase was 0.2 mL/min, and the sample was dried under a stream of air. The data were analyzed using LCMS solution analysis software 3.80.410.

### 4.6. Cellulose Binding Assay

The cellulose binding assay was performed as previously described [42]. Briefly, 0.2 mg of LPMO (10 mM HAc-NH4Ac buffer, pH 5.0) was incubated overnight with a series of concentrations of Avicel at 25 °C. After centrifugation (15,000 rpm, 30 min), the concentration of LPMO in the supernatant was measured by means of a BCA protein assay kit. The fraction of bound protein was determined as the difference between the initial concentration and the remaining concentration. The experiments were repeated three times independently. The adsorption constants were calculated by fitting the data to a Langmuir adsorption isotherm using Origin 8 software.

### 4.7. Molecular Dynamics (MD) Simulations

AutoDockTool 1.5.6 was used to perform molecular docking between the protein and ligands. The Amber 18 package was used for all MD simulations. Cellohexaose was used as a ligand for the analysis of the interaction between the substrate and AA9 LPMOs, whereas cellotetraose was used as the ligand for the analysis of the interaction between the product and AA9 LPMOs. The protein force field parameters and ligand force field parameters were supplied by ff14SB and gaff, respectively, and the ANTECHAMBER module was used to compute the AM1-BCC atomic charge. The MCPB module was used to compute the coordinating bonds that formed between the copper ion and histidine. The protein (and ligand) was constrained by heavy atoms, and 10,000 steps of energy minimization were performed for the water molecule. The MD simulation of the system was performed for 20 ns under the npt ensemble, and data were saved every other 5 ps. CPPTRAJ was used for data analysis. The MMPBSA.py module was used to compute the binding free energy between the protein and ligand.

### 4.8. Enzymatic Activity Assay

LPMO activity was confirmed as previously described [20]. Briefly, the reaction mixture included equal volumes of 1.2 μM Cu^2+^-*Ta*AA9, 800 μM rPHP, 400 μM DHA and citrate–phosphate buffer (pH 7.25), which were incubated for 30 min at 40 °C with shaking at 450 rpm. After that, 50 μL of stop buffer (Na_2_CO_3_, pH 10.3) was added to each well, and then absorption at 545 nm was measured. Replacing LPMO by bovine serum albumin (BSA) was used as the control. The experiments were repeated three times independently.

### 4.9. Synergy Assay

FPase (filter paper enzyme) and β-glucosidase activities were measured using previously published protocols [43]. PASC (5 mg/mL) and Avicel (30 mg/mL) were treated with LPMO according to the method described in the oxidative reaction product assay. After incubation, the supernatant was removed, and the precipitate was further incubated using the saccharification system. The composition of the saccharification system was based on our previously described method [40]. Briefly, the reaction system was 1mL of 50 mM sodium citrate at pH 4.8, which contained the cellulase and β-glucosidase with a 1:1 ratio based on FPase activity to β-glucosidase activity. The reaction was conducted at 45 °C for 24 h. The glucose content was determined using a high-performance liquid chromatography system (Hitachi, Ltd., Tokyo, Japan) as previously described [40]. The experiments were independently repeated three times.

### 4.10. Statistical Analysis

One-way analysis of variance was used to test the significant effects of variables, followed by Student’s *t*-test. *p*-values < 0.1 and 0.01 indicate significant differences.

## 5. Conclusions

Using computational and biochemical methods, we revealed the residue at position 2 of *Ta*AA9 that plays a key role in regulating the geometry coordinate of the copper and catalytic activity. We propose for the first time that the electropositivity around the copper center of AA9 LPMOs may exhibit a positive correlation with the enzymatic catalysis. Our study elucidates the effect of the key amino acid near the active site of AA9 LPMOs on their catalytic activities, allowing for a deep understanding of the catalytic mechanism of AA9 LPMOs.

## Figures and Tables

**Table 1:** The product profiles of *Ta*AA9, G2T-*Ta*AA9 and G2Y-*Ta*AA9.

Product Profiles	*Ta*AA9				G2T-*Ta*AA9				G2Y-*Ta*AA9			
	DP2	DP3	DP4	DP5	DP2	DP3	DP4	DP5	DP2	DP3	DP4	DP5
C1 (m/z + 16)	−	−	−	+	−	−	−	−	−	−	−	−
C4/C6 (m/z − 2)	+	−	+	−	+	+	+	−	+	+	+	−
C1 + C4 (m/z + 14; m/z + 32)	+	+	+	+	+	+	−	−	+	+	−	−
C1 + C6 (m/z + 30)	+	+	−	−	−	−	−	−	−	−	−	−
C4 + C6 (m/z − 4)	−	−	−	−	−	−	−	−	−	−	−	−
C1 + C4 + C6 (m/z + 28)	+	+	+	+	+	+	−	−	+	+	−	−

## Data Availability

The sequence of *Ta*AA9 LPMO can be found in the GenBank database at https://www.ncbi.nlm.nih.gov/genbank/ (accessed on 9 June 2009) with the accession No. ABW56451. The 3D structure of *Ta*AA9 LPMO can be found in the Protein Data Bank database at https://www.rcsb.org (accessed on 30 March 2011) with the accession ID: 2yet.

## Supplementary Materials

The following supporting information can be downloaded at: https://www.mdpi.com/article/10.3390/ijms24098300/s1.
