# Supplementary material for: The Role of the Residue at Position 2 in the Catalytic Activity of AA9 Lytic Polysaccharide Monooxygenases
Source: Int J Mol Sci. 2023 May 5;24(9):8300. doi: 10.3390/ijms24098300 (PMC10179388; doi:10.3390/ijms24098300)
Supplement: Supplementary file 1 [file ijms-24-08300-s001.zip › ijms-2318844-supplementary.pdf]

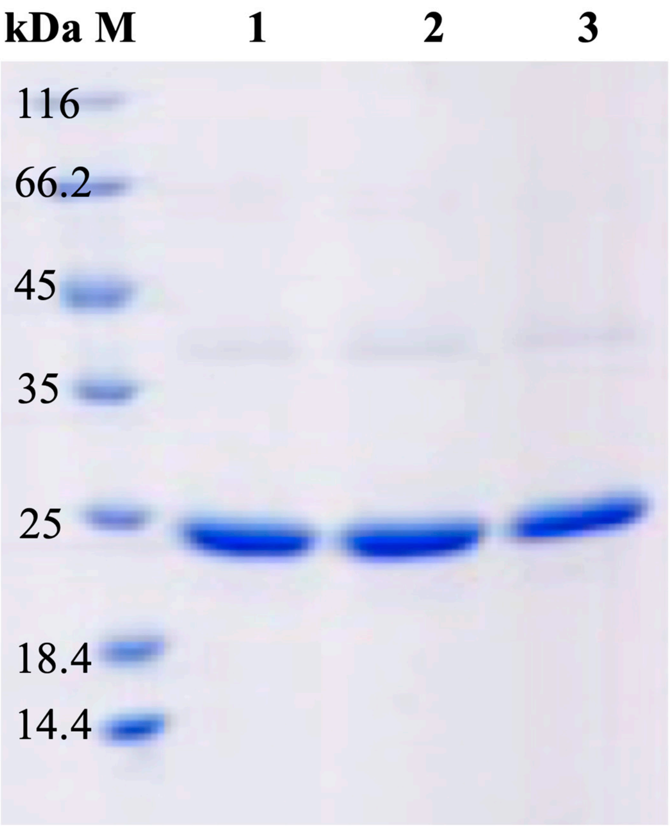

Figure S1. The recombinant *Ta*AA9 and its variant proteins were analyzed by SDS-PAGE followed by staining with Coomassie Blue R250. Lane M: protein marker; lane 1: *Ta*AA9; Lane 2: G2T-*Ta*AA9; Lane 3: G2Y-*Ta*AA9.

A

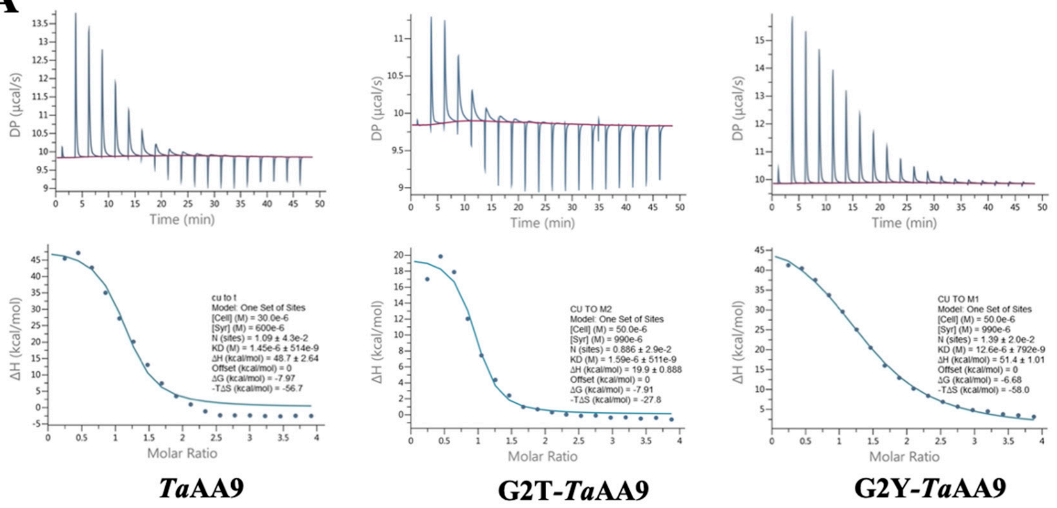

B

Protein	dissociation constant (Kd, M)
***Ta*AA9**	$1.45e^{-6}\pm514e^{-9}$
**G2T-*Ta*AA9**	$1.59e^{-6}\pm511e^{-9}$
**G2Y-*Ta*AA9**	$12.6e^{-6}\pm792e^{-9}$

Figure S2. Analysis of the affinity of copper to the catalytic center by the ITC assay.

(A) The titration curves of *Ta*AA9 and mutants as determined by ITC assay. (B)The dissociation constants for Cu^{2+} in *Ta*AA9 and its mutants. The assay buffer is 20 mM MES buffer, pH 5.5. Aliquots of 4 µL were injected at 150-s intervals with a stirring speed of 750 rpm at 25°C. The titrations were finished after 19 injections. The ITC data were analyzed using Microcal PEAQ-ITC analysis software

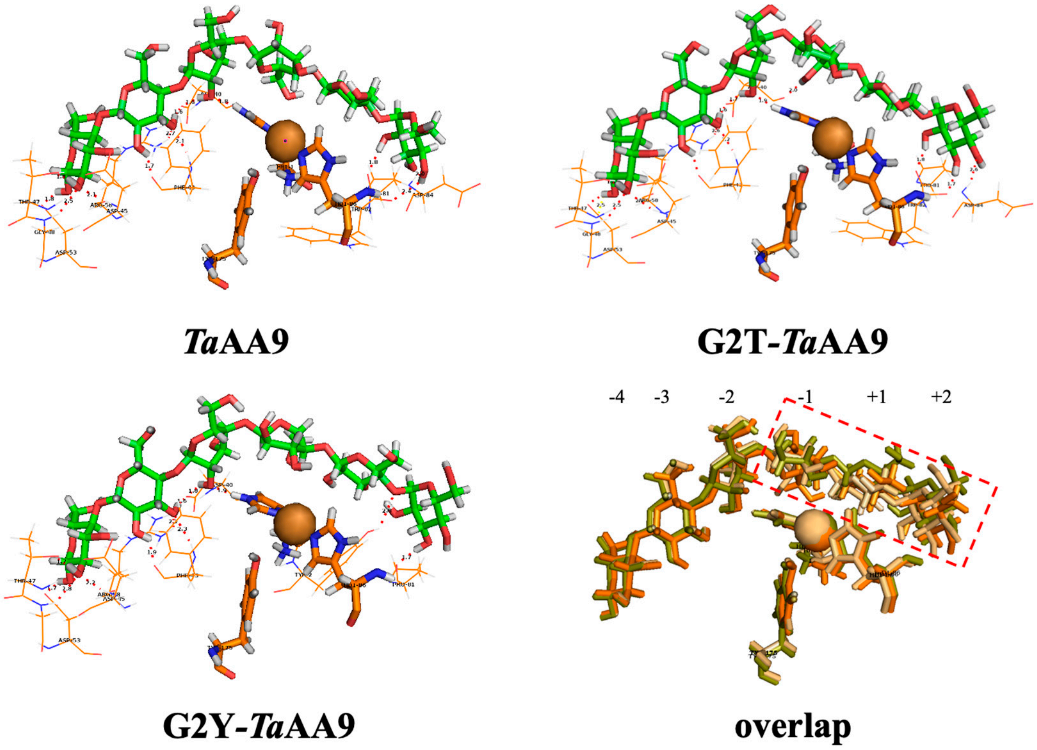

Figure S3. The binding mode of cellohexaose in *Ta*AA9A and its mutants. The overlap of the mode of cellohexaose in *Ta*AA9 and its mutants was visualized by PyMOL software. Orange: *Ta*AA9 (PDB ID: 2yet); Light orange: G2T-*Ta*AA9 and Yellow green: G2Y-*Ta*AA9. The sugar residues are numbered by subsite and the sugar closest to His1 is numbered as sugar +1. The red box indicated that the sugar residues (-1to +2) of cellohexaose were distorted when G2 was replaced with a thr or tyr residue.

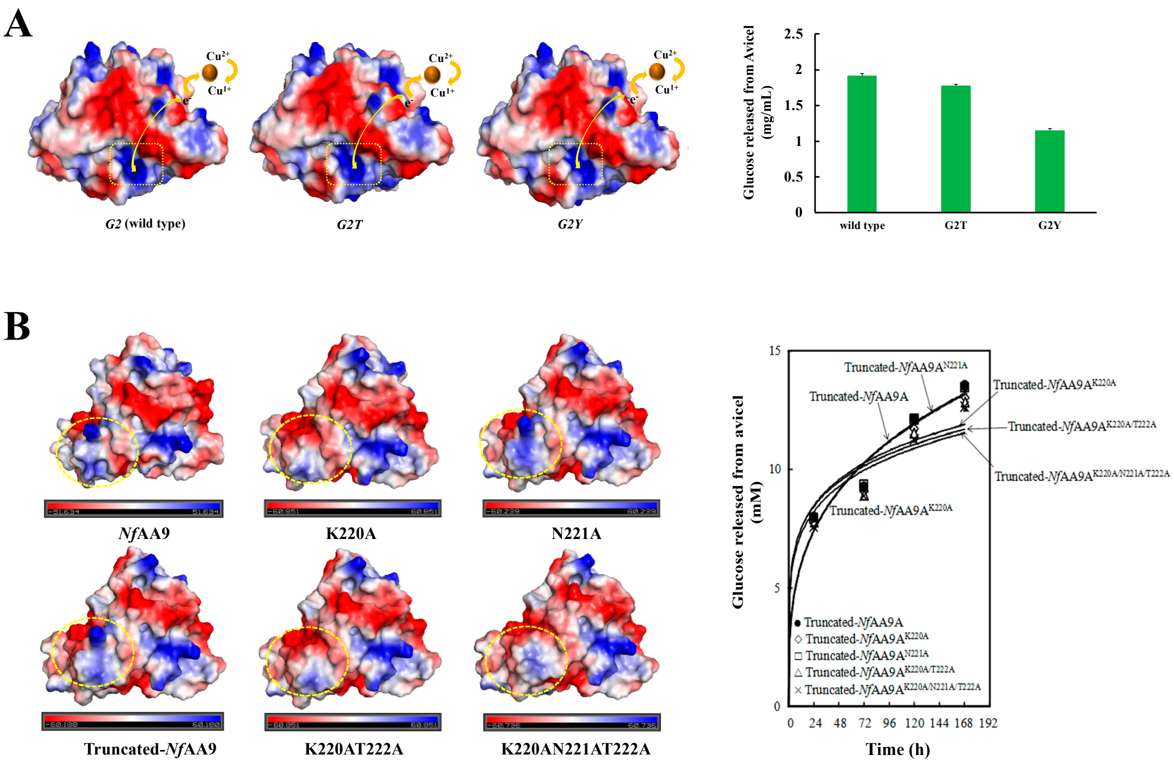

Figure S4. The analysis of surface charge and enzymatic activity of AA9 LPMO from *Trichoderma reesei* QM6A and other AA9 LPMOs. (A) ***Trichoderma reesei* QM6A; (B) other AA9 LPMO.** Left panels: analysis of surface charge of AA9 and mutants which was visualized by PyMOL software. Blue: electropositivity; Brown: copper ion; Yellow square: copper center.; Right histogram: the glucose quantity released from Avicel catalyzed by AA9 and cellulase. The reaction contained the cellulase and β-glucosidase (1:1 ratio) in 50 mM sodium citrate at pH 4.8. The glucose content was determined by HPLC. The data are presented as the mean ± standard deviation.

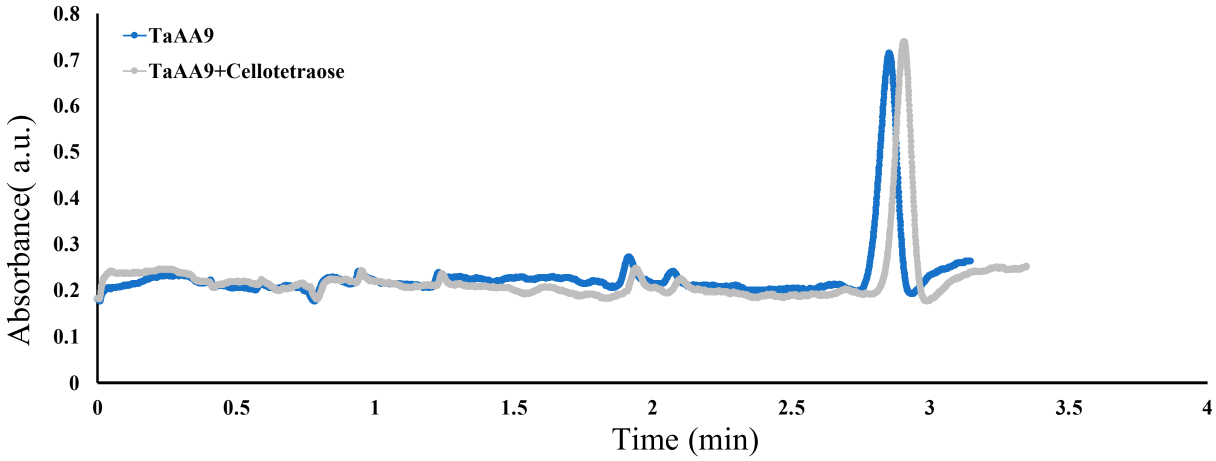

Figure S5. Interaction between *Ta*AA9 and cellotetraose measured by CE assay.

The migration time point of the *Ta*AA9 shifted as it interacted with cellotetraose.

A

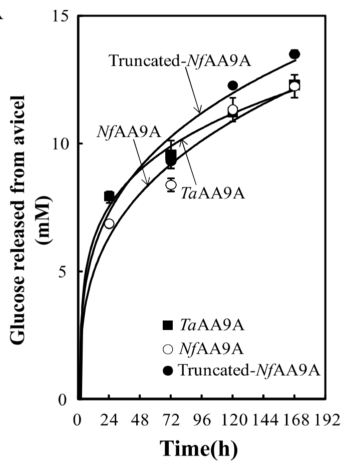

B

Protein	cellotriose	cellotetrose
***Nf*AA9**	-5.9065	-10.3604
**truncated-*Nf*AA9**	-2.7166	-4.2813

Figure S6. The analysis of enzymatic activity of AA9 LPMO and binding free energy between *Nf*AA9 or truncated-*Nf*AA9 and products. (A) Glucose released from Avicel by cellulase from *T. reesei* T1+β- glucosidase+AA9. (B) Binding free energy between *Nf*AA9 or truncated-*Nf*AA9 and the cellotriose or cellotetraose as determined by MD stimulations.

Table S1. The difference between two methods.

Experimental condition	Our experiment	Reference
Buffer	10mM sodium acetate	20 mM MES
pH	5	5.5
Expression host	*E.coli*	*A. oryzae*

Reference:

[1] Quinlan, R.J., Sweeney, M.D., Lo Leggio, L., Otten, H., Poulsen, J.C., Johansen, K.S., Krogh, K.B., Jørgensen, C.I., Tovborg, M., Anthonsen, A., Tryfona, T., Walter, C.P., Dupree, P., Xu, F., Davies, G.J., Walton, P.H. Insights into the oxidative degradation of cellulose by a copper metalloenzyme that exploits biomass components. *P Natl Acad Sci USA.* 2011. 108(37), 15079-84. doi: 10.1073/pnas.1105776108.

Table S2. The Km values of two methods.

Our experiment	Reference
249	244

Reference:

[1] Brander, S., Lausten, S., Ipsen, J.Ø., Falkenberg, K.B., Bertelsen, A.B., Nørholm, M.H.H, Østergaard, L.H., Johansen, K.S. Colorimetric LPMO assay with direct implication for cellulolytic activity. *Biotechnol Biofuel.* 2021. 14(1),51. doi:10.1186/s13068-021-01902-4.

Table S3. The Hydrogen bond Distance between amino acids and cellohexaose

**Protein**	**Hydrogen bond Distance between amino acids and substrate**											
	**H1**	**Y2**	**D40**	**F43**	**D45**	**T47**	**G48**	**D53**	**R58**	**P81**	**W82**	**D84**
***Ta*AA9**	1.8	–	1.6, 1.6	1.7, 2.1	2.1	2.7	2.5	1.8	2.7	1.8	2.4	2
***Ta*AA9^{G2T}**	1.9	–	1.6, 1.7, 2.0	1.8, 2.2	2.2	1.8	2.5	1.8	2.6	1.8	1.7	2.6
***Ta*AA9^{G2Y}**	1.9	2.8	1.6, 1.8	1.9, 2.3	2.2	1.7	2.8	1.7	2.7	1.7	–	–

Table S4. Binding free energy between *Ta*AA9 and mutants and the ligand as determined by MD stimulations

**Protein**	**Cellotetraose**	**Cellohexaose**
***Ta*AA9**	-3.7204	-26.2989
**G2T-*Ta*AA9**	-12.9768	-25.4728
**G2Y-*Ta*AA9**	-17.9968	-14.2602

Table S5. Primers used for site-directed mutagenesis

*Ta*AA9 variant	Primer pair (5'-3')
G2T	F: GGGTCATACCTTTGTGCAGAACATTGTGATTGACGGC
	R: GCAACTAAGCTCGCGCTCGCTAAAACG
G2Y	F: GGGTCATTATTTTGTGCAGAACATTGTGATTGACGGC
	R: GCAACTAAGCTCGCGCTCGCTAAAACG
